# Wie gelingt die Prävention psychischer Beschwerden? Von der Vorsorgeuntersuchung zur indikativen Präventionsmaßnahme bei emotionalen und Verhaltensauffälligkeiten im Vor- und Grundschulalter: eine prospektive Implementationsstudie

**DOI:** 10.1007/s11553-021-00838-9

**Published:** 2021-03-05

**Authors:** Max Weniger, Katja Beesdo-Baum, Veit Roessner, Helene Hense, Susanne Knappe

**Affiliations:** 1 Institut für Klinische Psychologie und Psychotherapie, Technische Universität Dresden, Dresden, Deutschland; 2 Institut für Klinische Psychologie und Psychotherapie, Behaviorale Epidemiologie, Technische Universität Dresden, Dresden, Deutschland; 3 Klinik und Poliklinik für Kinder- und Jugendpsychiatrie und -psychotherapie, Universitätsklinikum und Medizinische Fakultät Carl Gustav Carus, Technische Universität Dresden, Dresden, Deutschland; 4 Forschungsverbund Public Health Sachsen, Zentrum für Evidenzbasierte Gesundheitsversorgung (ZEGV), Universitätsklinikum und Medizinische Fakultät Carl Gustav Carus, Technische Universität Dresden, Dresden, Deutschland

**Keywords:** Emotionale und Verhaltensprobleme, Kinder und Jugendliche, Regelversorgung, Strength and Difficulties Questionnaire, Angstsymptome, externalisierendes Verhalten, Emotional and behavioral problems, Children and adolescents, Standard care, Strength and Difficulties Questionnaire, Anxiety symptoms, Externalizing behaviors

## Abstract

**Hintergrund:**

Emotionale und Verhaltensprobleme in der Kindheit haben oft weitreichende Folgen für die soziale, emotionale und kognitive Entwicklung, sodass ihrer Prävention ein hoher Stellenwert zukommt. Dennoch ist die Inanspruchnahme von Präventionsmaßnahmen im Kindesalter gering.

**Ziel:**

In einer versorgungsepidemiologischen Implementationsstudie wird untersucht, inwiefern durch ein systematisches Screening eingebettet in U‑Untersuchungen die Identifikation von Risikokindern und die Zuweisung zu Präventionsprogrammen gelingt.

**Methoden:**

Dazu ist ein Screening mittels „Strengths and Difficulties Questionnaire“ während der regulären Gesundheitsuntersuchungen (U9–U11) von ca. 3500 Kindern im Alter von 5 bis 10 Jahren in ca. 53 Arztpraxen in Dresden und 20 km Umkreis geplant. Die Eltern erhalten von der Fachkraft für Kinderheilkunde eine Rückmeldung zu den Ergebnissen und im Falle von grenzwertigen Werten auf den Subskalen „Emotionale Probleme“ und/oder „Verhaltensprobleme“ eine Empfehlung für ein indikatives Präventionsprogramm. Zu vier Messzeitpunkten werden Familien mittels standardisierter und projektspezifischer Fragebogen befragt. Zusätzlich erfolgen leitfadengestützte Interviews mit Leistungserbringern und Familien.

**Ergebnisse und Schlussfolgerung:**

Es werden die Machbarkeit, Nützlichkeit und Akzeptanz eines Screenings für emotionale und Verhaltensauffälligkeiten bei Kindern und Präventionsempfehlungen in Kinderarztpraxen im Prä‑/Post-Vergleich und nach 12 Monaten evaluiert. Förderliche und hemmende Faktoren für die Inanspruchnahme werden bestimmt, um Empfehlungen für die Implementation von Präventionsangeboten in die Regelversorgung abzuleiten, um emotionale und Verhaltensauffälligkeiten frühzeitig zu erkennen und der Entwicklung psychischer Störungen vorzubeugen.

## Abstract (en)

**Background:**

Emotional and behavioral problems in childhood often have far-reaching consequences for social, emotional and cognitive development, which is why their prevention is of great importance. However, preventive measures during childhood are rarely taken.

**Objectives:**

As part of a health care implementation study, it will be examined whether systematic screening, embedded in routine pediatric health check-ups promotes the identification of children at risk and the allocation to preventive programs.

**Methods:**

For this purpose, a screening via the Strengths and Difficulties Questionnaire is planned during the regular health check-ups (U9–U11) of approximately 3500 children aged 5–10 years in about 53 medical practices within a 20 km radius of Dresden (Germany). The parents receive feedback concerning the results from the pediatrician and, in case of borderline results on the subscales “emotional problems” and “behavioral problems”, a recommendation for an indicative prevention program. Children and parents will be assessed at four points in time with standardized and project-specific questionnaires. Furthermore, guided interviews with the health professional and the parents will be conducted.

**Results and conclusions:**

The feasibility, utility and acceptance of a screening for emotional and behavioral problems of children and recommendations for their prevention in pediatricians’ practices will be evaluated in a pre–post comparison and after 12 months. Beneficial and inhibiting factors for the utilization are to be identified and used to derive recommendations for the implementation of preventive measures in standard care, identify emotional and behavioral problems at an early stage and prevent the development of mental disorders.

Da sich die Mehrzahl psychischer Störungen, allen voran Angst-, depressive und verhaltensbezogene Störungen, bereits im Kindes- und Jugendalter entwickeln, bestimmen sie maßgeblich die soziale, emotionale und kognitive Entwicklung. Indikative Präventionsprogramme können im Vergleich zu universellen Maßnahmen eine bedeutsame Symptomreduktion bewirken. Allerdings ist die Inanspruchnahme solcher häufig auch überzeugend evaluierter Programme in der Routineversorgung bislang gering.

Das PROMPt-Projekt (www.prompt-projekt.de) hat zum Ziel, ein Screening für die Identifikation von Kindern mit emotionalen und Verhaltensproblemen und die Zuweisung zu indikativen Präventionsprogrammen zu implementieren und zu evaluieren. Zusätzlich sollen Barrieren und Prädiktoren für die Inanspruchnahme von Präventionsprogrammen identifiziert und daraus Maßnahmen abgeleitet werden, um zukünftig eine optimierte Versorgungskette in der Routineversorgung zu schaffen und die Inanspruchnahmerate zu erhöhen.

## Hintergrund und Fragestellung

Unter den 3- bis 6‑jährigen Kindern leiden 6,6 % bzw. 15,8 % und unter den 7- bis 10‑Jährigen 10,4 % bzw. 15,1 % an emotionalen oder Verhaltensauffälligkeiten [14, 21]. Unbehandelt können emotionale und Verhaltensprobleme zu erheblichen Beeinträchtigungen führen und Entwicklungspfade maßgeblich ungünstig beeinflussen [3, 13]. Frühzeitig angebotene Präventionsmaßnahmen könnten einer Manifestation psychischer Störungen entgegenwirken und damit mit hoher Wahrscheinlichkeit enorme Kosten im Gesundheits- und Sozialwesen, wie langwierige Behandlungskosten, Einbußen durch eine geringere Teilhabe und eingeschränkte Produktivität im Arbeitsleben reduzieren [17].

Wenngleich universelle Präventionsmaßnahmen (u. a. zur Zahngesundheit, Schuleingangsuntersuchungen) gesellschaftlich auf hohe Akzeptanz stoßen, bleiben Präventionsmaßnahmen zur psychischen Gesundheit und Gesundheitsförderung unterrepräsentiert. So sind derzeit eine Vielzahl von Präventionsangeboten für verschiedene Altersklassen und Störungsbereiche verfügbar; verhaltenspräventive Angebote der Krankenkassen werden aber nur von 3 % der unter 20-Jährigen genutzt [4]. Angesichts dieser geringen Inanspruchnahme verfügbarer und wirksamer Präventionsmaßnahmen bei zugleich hohen Raten für Verhaltensauffälligkeiten und psychischen Störungen sollen modellhaft im Rahmen einer neuen Versorgungsform die Zuweisung zu indikativen Präventionsprogrammen erprobt sowie hinderliche und förderliche Faktoren für die Inanspruchnahme identifiziert werden.

## Studiensdesign und Untersuchungsmethoden

**Abb. 1:**
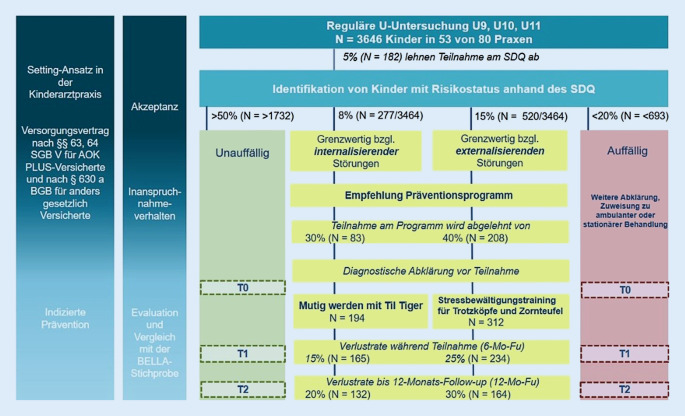
PROMPt-Studiendesign und geplante Fallzahlen (*SDQ* Strengths and Difficulties Questionnaire, *Fu* Follow-up, *T0-T2*  Messzeitpunkte)

Das Projekt *PROMPt *(Primärindikative und optimierte Zuweisung zu gezielten Maßnahmen bei emotionalen und Verhaltensauffälligkeiten bei Kindern, www.prompt-projekt.de) ist eine prospektive Implementationsstudie zur Evaluation der Machbarkeit, Nützlichkeit und Akzeptanz eines Screenings für emotionale und Verhaltensauffälligkeiten bei Kindern von 5 bis 10 Jahren in der primärärztlichen Versorgung durch Fachärztinnen und Fachärzte der Kinder- und Jugendmedizin (FfPaed) in Dresden. Zudem sollen hemmende und Gelingensfaktoren für die Zuweisung zu und Teilnahme an einer indikativen Präventionsmaßnahme bestimmt werden. Zur Evaluation des Versorgungsverlaufs erfolgen Befragungen nach dem Screening sowie nach 6 Monaten (bzw. nach der Intervention) und 12 Monaten (Abb. 1). Qualitative Interviews mit Familien und Leistungserbringern erfassen individuelle Erfahrungen zum Versorgungsverlauf. Ferner erfolgt ein Vergleich zur Pair-matched-BELLA-Studie zur Gesundheit von Kindern und Jugendlichen [14], um die längerfristige behaviorale und emotionale Entwicklung zwischen Kindern mit und ohne Teilnahme an einem Präventionsprogramm zu vergleichen.

### Vorgehen

Im Rahmen der regulären Gesundheitsuntersuchungen (U-Untersuchungen, U9: 5–6 Jahre, U10: 7–8 Jahre, U11: 9–10 Jahre) werden Kinder anhand des „Strengths and Difficulties Questionnaire“ (SDQ; [10]) von ihrem gesetzlichen Vertreter (zumeist einem Elternteil) zu Verhaltensauffälligkeiten und -stärken eingeschätzt. Die Einschätzung findet während der Wartezeit statt und ist freiwillig. Das Praxispersonal gibt die Fragebogenmappe an den gesetzlichen Vertreter aus. Um die ärztliche Routine nicht maßgeblich zu unterbrechen, wird der ausgefüllte SDQ entweder vom Praxispersonal oder von einer FfPaed mittels Schablone ausgewertet. Gemäß den erzielten Werten werden die Kinder zu keiner Intervention, zu einem etablierten indikativen Präventionsprogramm oder zur weiteren Abklärung an regionale Ansprechpartner verwiesen. Die Empfehlung für ein indikatives Präventionsprogramm erfolgt, wenn ein Schwellenwert für internalisierende (Rohwert von 4 auf der SDQ-Skala „Emotionale Probleme“) und/oder externalisierende Verhaltensprobleme (Rohwert von 3 auf der SDQ-Skala „Verhaltensprobleme“) erreicht ist oder die FfPaed aufgrund ihrer Expertise eine Empfehlung ausspricht. Die Familien erhalten im Falle einer Präventionsempfehlung eine Broschüre mit Angaben zu wohnortnahen Präventionsangeboten und Ansprechpartnern sowie zur Kostenübernahme. Bei diagnostischer Unsicherheit oder überschwelligen Werten auf den relevanten SDQ-Skalen erhalten die gesetzlichen Vertreter Kontaktdaten von geeigneten Einrichtungen oder eine Überweisung in die ambulante/stationäre Krankenversorgung für die Möglichkeit einer genaueren Abklärung.

Die FfPaed erhalten für die zusätzliche Leistung (Durchführung, Auswertung und Ergebnisrückmeldung zum SDQ an die gesetzlichen Vertreter) 8 € pro Kind, orientiert an vergleichbaren Leistungen der EBM-Vorgaben. Für AOK PLUS versicherte Kinder geschieht dies auf Basis eines Versorgungsvertrags nach §§ 63, 64 SGB V (Modellvorhaben) zwischen den FfPaed und der AOK PLUS. Für Patienten anderer gesetzlicher Krankenkassen erhalten sie die Aufwandsentschädigung auf Grundlage des § 630a BGB.

### Stichprobe

Es sollen mindestens 53 FfPaed zur Teilnahme gewonnen werden. Bemessen auf die Bevölkerungszahl Dresdens (Stand 09/2017) wird von einer Grundgesamtheit von 5503 Kindern im Alter von 5 bis 10 Jahren ausgegangen, von denen 85 % (*n* = 3646/5503) aller gesetzlich versicherten Kinder nach Schätzungen der allgemeinen Ortskrankenkasse für Sachsen und Thüringen an einer U9-, U10- oder U11-Untersuchung teilnehmen. Ausgehend von einer Häufigkeit von 7,9 % für emotionale und 14,8 % für Verhaltensprobleme [14] und einer Ablehnungsquote gegenüber dem Screening von 5 % können 277 (emotionale Auffälligkeiten) bzw. 520 (Verhaltensauffälligkeiten) Kinder positiv gescreent werden und eine Empfehlung zur Teilnahme am Präventionsprogramm erhalten (Abb. 1). Unter der Annahme unterschiedlicher Adhärenz- und Verlustraten je Problembereich (30 % bzw. 40 %) werden konservativ geschätzt in die Intervention 194 bzw. 312 Kinder/Familien eingeschlossen. Für die Posterhebung (12-Monats-Nacherhebung) sollen 165 (132) bzw. 234 (164) Kinder erreicht werden. Diese Fallzahlen würden zugleich ausreichen, zu erwartende mittlere Effekte auf Symptomebene durch die indizierten Präventionsprogramme [2, 6, 8] auf einem einseitigen Signifikanzniveau von *p* =0,05 und einer Power von 0,8 abzubilden. Konkret müssten 139 Teilnehmer pro Präventionsprogramm eingeschlossen werden, um einen Effekt von 0,3 bei einer Teststärke von 0,8 und A‑priori-Signifikanz von 0,05 zu zeigen.

Familien, die nicht wegen einer U‑Untersuchung bei ihrer FfPaed vorstellig sind, jedoch Interesse an dem Projekt haben, können, sofern die nächste U‑Untersuchung im nächsten halben Jahr stattfindet, ebenfalls über ihre FfPaed am Screening teilnehmen. Wenn die FfPaed nicht an dem Projekt teilnimmt oder die U‑Untersuchung erst nach einem halben Jahr stattfindet, können Familien über alternative Zugangswege am Präventionsprogramm teilnehmen (z. B. Selbstzuweiser). Diese alternativen Zugangswege werden beobachtet, jedoch nicht gezielt befördert.

Im Falle einer Präventionsempfehlung und Kontaktaufnahme der Familie mit dem Studienteam findet ein Vorgespräch mit Studienmitarbeitenden, mindestens einem gesetzlichen Vertreter und dem Kind statt, um diagnostisch zu klären, ob das Kind von einem Programm profitieren kann. Kinder können am Programm nur dann teilnehmen, wenn neben der Einwilligung der Sorgerechtstragenden auch ihr Einverständnis vorliegt. Ausschlusskriterien für eine Teilnahme am Präventionsprogramm sind eine bekannte aktuelle psychische Störung nach ICD-10 beim Kind, eine aktuelle psychotherapeutische Behandlung, akute Suizidalität oder eine instabile Medikation.

### Präventionsmaßnahmen

Je nach Symptomatik findet eines von zwei empirisch fundierten indikativen Präventionsprogrammen Anwendung, die nach § 20 Abs. 1 SGB V als theorie- und evidenzbasierte Frühpräventionsmaßnahmen mit kognitiv-behavioraler Ausrichtung zum multimodalen Stressmanagement anerkannt und somit Teil der Regelversorgung sind. Dies ist zugleich die sozialrechtliche Grundlage für die Erstattung der Teilnahmegebühren durch die meisten gesetzlichen Kranken- und Ersatzkrankenkassen. Einige Krankenkassen sehen auch den Einsatz eines Präventionsgutscheines vor, damit die Familien die Teilnahmegebühren nicht verauslagen müssen.

Das indikative Präventionsprogramm* Mutig werden mit Til Tiger* [2] zielt auf sozial ängstliche, schüchterne und gehemmte Kinder ab. Schrittweise werden in 2 Einzel- und 9 Gruppenstunden Selbstbewusstsein, der Umgang mit Stress und Gefühlen vermittelt sowie praktische Handlungsstrategien für den Alltag eingeübt. Ziel ist auch, den Risikofaktor „soziale Unsicherheit“/„Schüchternheit“ zu verändern und damit die Erkrankungswahrscheinlichkeit für Soziale Ängste (Phobien) maßgeblich zu mindern.

Das *Stressbewältigungstraining für Trotzköpfe und Zornteufel *basiert auf dem *Baghira-Gruppentraining* als Präventionsprogramm für Kinder mit oppositionellem und aggressivem Verhalten [1] und umfasst 9 Gruppenstunden sowie begleitende Elternarbeit, in denen mit den Kindern Strategien zur Wut- und Ärgerkontrolle sowie zur angemessenen Konfliktlösung erarbeitet und in verschiedenen Situationen und Rollenspielen vertieft werden. Durch ein Belohnungsprogramm wird das erwünschte Verhalten im Verlauf des Trainings gefestigt. Das Programm wurde ursprünglich für Kinder von 8 bis 13 Jahren konzipiert. Leichte Modifikationen ermöglichen auch den Einsatz bei jüngeren Kindern.

Die Wirksamkeit von *Mutig werden mit Til Tiger* wurde in einem Wartekontrollgruppendesign erfolgreich im Hinblick auf eine Steigerung des Selbstwerts, der Sozialkontakte sowie eine Reduktion der Unsicherheit und der Anzahl von Kindern mit klinisch relevanten sozialen Ängsten evaluiert [15]. Auch für das *Baghira-Training* [1] wurde im ambulanten und stationären Bereich eine deutliche Verminderung des aggressiven und delinquenten Verhaltens gezeigt.

### Evaluation des Versorgungspfades

Die Beurteilung des Versorgungspfades in die indikative Prävention erfolgt während der U‑Untersuchung als Screening (S), vor (T0) und nach dem Präventionsprogramm (T1), sowie an einer 12-Monats-Nacherhebung (T2; Tab. 1). Für Kinder, die im Screening unauffällige bzw. hoch auffällige Werte erreichen, ist eine Befragung zur Erhebung des Entwicklungsverlaufes direkt nach dem Screening (T0) sowie eine Nacherhebung nach 6 (T1) und 12 Monaten (T2) geplant. Eltern, die nicht am Projekt oder am Training teilnehmen möchten bzw. abbrechen, erhalten einen Nicht-Teilnahmefragebogen, um die Gründe dafür zu erfahren.

**Tab. 1:** Quantitative und qualitative Datenerhebung – Konstrukte, Messinstrumente und -zeitpunkte

Kategorie/Konstrukt	Messinstrument^b^	Messzeitpunkt^a^						
		Alle^E^	Risikogruppe^E, K^			Hoch-/unauffällige^E^		
		S	T0	T1	T2	T0	T1	T2
*Soziodemographische Angaben*	Projektspezifische Items	X	X	X	X	X	X	X
*Barrieren*								
Inanspruchnahme von Hilfe; (Nicht‑)Teilnahme an der Studie oder einem Präventionsprogramm; bei Studien- oder Programmabbruch; Stigmatisierung^E^	Projektspezifische Items	**	–	–	–	–	–	–
*Seelische Gesundheit des Kindes*								
Funktionalität und Lebensqualität^E, K^	[20]	–	X	X	X	X	X	X
Depressivität^E, K^	[5]	–	X	X	X	X	X	X
Ängstlichkeit^E, K^	[18]	–	X	X	X	X	X	X
Emotionale und Verhaltensprobleme^E^	[10]	X	–	X	X	–	X	X
Störung des Sozialverhaltens^E^	[7]	–	X	X	X	–	–	–
Skala „sozial-emotionale Kompetenzen“ des DISYPS^E^	[7]	–	X	X	X	X	X	X
Regelverletzendes, aggressives Verhalten^E^	[11]	–	X	X	X	X	X	X
Frühkindliche Verhaltensweisen^E^	[9]	–	X	–	–	–	–	–
Furcht^K^	[19]	–	X	X	X	–	–	–
Emotionen erkennen/regulieren; soziale Situationen verstehen^K^	[12]	–	X	X	X	–	–	–
*Elterliches seelisches Befinden*								
Elterliche psychische Störungen^E^	[26]	–	X	–	–	–	–	–
Belastung und Beeinträchtigung der Eltern^E^	[16]	–	X	X	X	X	X	X
Stressbelastung^E^	[25]	–	X	X	X	X	X	X
*Weitere Elternangaben*								
Erziehungsstil^E^	[22]	–	X	–	–	X	–	–
Familienklima^E^	[23]	–	X	–	–	X	–	–
Teilnahmemotivation^E^	Projektspezifische Items	–	X	–	–	–	–	–
Adhärenz^E^	Projektspezifische Items	–	–	X	–	–	–	–
Gesundheitskompetenz der Eltern^E^	[24]	–	X	–	–	–	–	–
Inanspruchnahme von Gesundheitsleistungen^E^	Projektspezifische Items	X	–	–	X	–	–	X
*Dokumentation des finanzbezogenen Aufwands*^E^ (Zahlungsmodalitäten etc.)	Projektspezifische Items	–	–	X	X	–	–	–
*Kursangaben*	Projektspezifische Items	–	X	X	–	–	–	–
*Angaben der FfPaed*	Projektspezifische Items	–	–	–	–	–	–	–
Empfehlung der FfPaed^A^	–	X	–	–	–	–	–	–
Alter, Geschlecht^A^	–	***	–	–	–	–	–	–
Anzahl Mitarbeitende in der Praxis, Weiterbildungen^A^	–	***	–	–	–	–	–	–
Umgang mit psychischen Auffälligkeiten bei Kindern^A^	–	***	–	–	–	–	–	–
Berufstätigkeit (Profession), Berufserfahrung in Jahren^A^	–	*	–	–	–	–	–	–
*Evaluation*								
Mittel- bis langfristige Evaluation der Trainingseffekte (Belastungsreduktion, Wirksamkeit der Programme)^E, K, T^	Projektspezifische Items	–	–	X	X	–	–	–
Evaluation der Trainer (Qualität des Programms, Kompetenz des Kursleiters, Zufriedenheit mit dem Programm)^E, K^	Projektspezifische Items	–	–	X	X	–	–	–
Machbarkeit des Vorhabens^E, A^	Interviewleitfaden	*	–	–	–	–	–	–
Akzeptanz des Vorhabens^E, A^	Interviewleitfaden	*	–	–	–	–	–	–
Nützlichkeit des Vorhabens^E, A^	Interviewleitfaden	*	–	–	–	–	–	–
*Fragen zum Versorgungsverlauf*^*E*^	Projektspezifische Items	–	–	–	–	–	X	X

^a^ *S* = Screening (während der regulären Gesundheitsuntersuchung), *T0* = Prä-Untersuchung bzw. äquivalente erste Erhebung bei unauffälligen/hoch auffälligen Kindern, *T*1 = Post-Untersuchung bzw. äquivalente zweite Erhebung bei unauffälligen/hoch auffälligen Kindern, *T2* = 12-Monate-Follow-up (12 Monate nach T0)

* = konsekutiver Einschluss, ** = bei Nicht-Teilnahme oder Abbruch, *** = bei Praxisrekrutierung/-einweisung

^b^ Beurteiler: *E* Eltern; *K* Kind, *A* FfPaed (Arzt); *T* Trainer (Präventionsprogramme)

Neben dem SDQ werden während der U‑Untersuchung individuelle, familiäre und versorgungstechnische Barrieren und Prädiktoren für die Zuweisung und Inanspruchnahme von Präventionsprogrammen zur Förderung der seelischen Gesundheit erfragt, z. B. Gründe einer (Nicht‑)Teilnahme, Einstellungen, Inanspruchnahmeverhalten von Hilfen und Gesundheitsleistungen. Für die mittel- und langfristige Evaluation der Trainingseffekte werden die Qualität und die Zufriedenheit mit dem Programm sowie die subjektive Wirksamkeit der Programme erfragt. Zusätzlich werden die Programmtrainer von den Kindern und ihren Eltern hinsichtlich der Kompetenz und des Umgangs mit den Kindern bewertet.

Ergänzend werden 16 Familien und 8 FfPaed/Praxispersonal qualitativ mittels leitfadengestützter Einzelinterviews zu ihren Erfahrungen mit dem SDQ-Screening in der Kinderarztpraxis, zu Zugangswegen zum Training, der Zuweisung zu und Durchführung des Programms sowie zu Aufwand und Zufriedenheit mit dem Gesamtprojekt befragt.

Primäre Ergebnismaße zur Evaluation des Versorgungspfades sind Teilnahmeraten am Screening, Adhärenz zum Versorgungsverlauf und zum jeweiligen Präventionsprogramm, Teilnahmemotivation und Drop-out-Raten.

Sekundäre Ergebnismaße sind Angaben zur behavioralen und psychischen Entwicklung der Kinder [7, 9, 11, 12, 18], zu frühkindlichen Verhaltensweisen [20] sowie zum Funktionsniveau und der Lebensqualität [26], Angaben zur seelischen Befindlichkeit und Gesundheitskompetenz der Eltern [16, 24, 25] sowie zur Inanspruchnahme von Gesundheitsleistungen, zum Erziehungsstil [22] und zum Familienklima ([23]; Tab. 1).

Die Rekrutierung der FfPaed startete Ende 01/2020 und ist für 12 Monate geplant. Der Rekrutierungsverlauf gestaltet sich zufriedenstellend, trotz zeitgleicher Quartals- und Jahresabrechnung in den Arztpraxen, jahreszeitlich bedingter erhöhter Anzahl von Erkältungs- und Grippefällen und damit Mehrbelastungen der FfPaed. Die Einweisung der ersten Praxen und erste Screenings wurden Ende 02/2020 durchgeführt. Der Einschluss des ersten Kindes in eines der Präventionsprogramme war zum 03/2020 für 12 Monate geplant. Der Einschluss des letzten Teilnehmers in das Screening während einer regulären Gesundheitsuntersuchung bei der FfPaed ist für Ende des 2. Quartals 2021 vorgesehen. Das Projekt wurde aufgrund der COVID-19-Pandemie und der Schließung des Universitätsbetriebs unterbrochen und zum 25.05.2020 schrittweise wiederaufgenommen.

## Diskussion

Für das PROMPt-Projekt wird ein nachhaltiger Nutzen auf unterschiedlichen Ebenen erwartet: Unmittelbar profitieren die teilnehmenden Kinder und Familien, indem eine frühzeitige Diagnostik, eine damit verbundene Empfehlung und gegebenenfalls Teilnahme an einem etablierten indikativen Präventionsprogramm erfolgt. Mittelfristig werden erstmals und spezifisch für Dresden und Umgebung am Beispiel von emotionalen und Verhaltensauffälligkeiten Hürden und Gelingensfaktoren für die Identifikation von Risikopersonen, ihre Zuweisung zu und Teilnahme an einer indikativen Präventionsmaßnahme bestimmt. Ferner werden durch die qualitativen Analysen Aussagen zur Umsetzbarkeit des Programms (u. a. Aufwand, Zufriedenheit, Nützlichkeit) aus Sicht der FfPaed und Versicherten möglich.

Langfristig besteht das Ziel in der routinemäßigen, bevölkerungsbasierten Identifikation von Risikogruppen und der Senkung der Inzidenz psychischer Störungen über die Region hinaus durch gezielte Prävention und Frühintervention. Schlussendlich werden Erkenntnisse darüber gewonnen, welche Hürden bei der Umsetzung bedeutend sind, was zukünftig verbessert und worauf bei einer Optimierung der Versorgungskette geachtet werden muss.

## Schlussfolgerungen

Die niederschwellige Intervention nutzt Maßnahmen, die praktikabel, einfach und kostengünstig sind und die durch Öffentlichkeitswirksamkeit und Dissemination nachhaltig zu einer verbesserten Situation und Entstigmatisierung im Umgang mit psychischen Beschwerden beitragen können. Durch die vertragliche Ausgestaltung wird eine Versorgungskette erprobt und schafft bestenfalls eine neue Form der Regelversorgung in Form einer Ausweitung der Vorsorgeuntersuchungen auf psychische Erkrankungen, die Vermeidung von Folgekosten und Erhöhung der Lebenseinstiegschancen.

## Fazit für die Praxis

- Die Inanspruchnahme von Präventionsprogrammen zur Förderung der seelischen Gesundheit ist alarmierend gering – bei zugleich hohen Raten von Verhaltensauffälligkeiten und psychischen Störungen.
- Die Schaffung einer optimierten Versorgungskette in der Routineversorgung und Ausweitung der kinderärztlichen Vorsorgeuntersuchung auf psychische Erkrankungen kann möglicherweise die Zuweisung und Inanspruchnahme indikativer Präventionsprogramme erhöhen.
- Langfristig können durch eine frühzeitige Identifikation Betroffener einer Manifestation psychischer Störungen entgegenwirkt, die Inzidenz psychischer Störungen gesenkt und mit hoher Wahrscheinlichkeit enorme Kosten im Gesundheits- und Sozialwesen reduziert werden.
